# What practical strategies improve recruitment and engagement of people experiencing homelessness in observational clinical research? A multistudy synthesis from Dublin, Ireland

**DOI:** 10.1136/bmjopen-2025-114055

**Published:** 2026-03-26

**Authors:** Georgia Richard, Conor Reddy, Claire Lyons, Ruth Argue, Nollaig M Bourke, Clíona Ní Cheallaigh

**Affiliations:** 1 Department of Neurology, St James’s Hospital, Dublin, Ireland; 2 Discipline of Clinical Medicine, Trinity College Dublin, Dublin, Ireland; 3 Independent Researcher, Dublin, Ireland; 4 Clinical Research Facility, St James’s Hospital, Dublin, Ireland; 5 Discipline of Medical Gerontology, Trinity College Dublin, Dublin, Ireland; 6 Clinical Medicine, Trinity College, Dublin, Dublin, Ireland; 7 General Medicine, St James’s Hospital, Dublin, Dublin, Ireland

**Keywords:** Research Design, Vulnerable Populations, SOCIAL MEDICINE

## Abstract

**Abstract:**

**Objectives:**

This study aims to identify practical, trauma-informed strategies to improve engagement of populations experiencing homelessness in observational health research.

**Design:**

Data from three real-world observational studies involving people experiencing homelessness (PEH) and housed participants were analysed for study completion rates and participant demographics. A team of researchers, clinicians and people with lived experience of homelessness, reviewed their experience of study design, recruitment and assessment study procedures to identify strategies found to be effective in recruitment and retention of PEH.

**Setting:**

The Inclusion Health Research Group (IHRG) consists of 10 clinicians and researchers who study the effect of social exclusion, such as homelessness, on health. Many members of the group also provide clinical care to PEH. The three observational studies which informed this paper recruited participants in Dublin, Ireland between 2021 and 2025. Approximately 15 000 adults are currently experiencing homelessness in Ireland, of whom two-thirds reside in Dublin and more than 70% are accommodated in hostels.

**Participants:**

The three observational studies that informed this paper, designed and carried out by members of the IHRG, included populations of PEH recruited from sites in the community across Dublin.

**Results:**

PEH were predominantly male with an average age in their mid-40s and a high prevalence of smoking, drug use and alcohol consumption. Study completion rates were high (83.3% to 94.1%). On review of the study design and implementation, eight key strategies were identified as effective in recruitment and retention of PEH: (1) involvement of people with lived experience in study design; (2) flexible recruitment locations; (3) flexible assessment timings; (4) trust-building through consistent communication; (5) collaboration with gatekeeper organisations and peer advocates; (6) use of non-financial incentives like health screenings; (7) validated assessment tools and (8) tailored support for participants with addiction.

**Conclusions:**

Using flexible, trauma-informed, participant-centred approaches informed by the involvement of people with lived experience and gatekeeper organisations can ensure high levels of recruitment and retention of PEH. This may enhance research inclusivity and ultimately contribute to better understanding of the complex health needs of populations experiencing homelessness within public health.

**STRENGTHS AND LIMITATIONS OF THIS STUDY**

- Addresses gaps in existing evidence on recruitment and retention of people experiencing homelessness (PEH) in observational health research and supports previously published existing evidence.
- Synthesises recruitment/retention strategies across three real-world observational studies with high rates of recruitment and retention of multiply excluded adults experiencing homelessness in Dublin, Ireland.
- Includes multidisciplinary evaluation with direct input from an expert-by-experience (lived experience of homelessness) to support coproduced interpretation, although as only a single author with lived experience contributed to the work, validation and member checking of PEH with diverse backgrounds were not performed.
- Themes were developed through team-based synthesis rather than a formal qualitative study design (eg, a modified Delphi process), which may limit reproducibility and transferability.
- Findings are context-specific to one research group and one urban Irish setting, which may limit generalisability to other systems and populations.

## Background

 Social exclusion, defined as the involuntary non-participation in any combination of social, cultural, educational, economic and/or political activities in society and poverty, drives the accumulation of allostatic load (the cumulative physiological burden of chronic stress on the body)^1^ and results in adverse health outcomes and increased mortality.^1^,^5^ People experiencing homelessness (PEH) can be considered the section of society in whom the cumulative effects of allostatic load resulting from lifelong adversity, intergenerational trauma and social exclusion are maximally seen.^6^ This is reflected in the high rates of multimorbidity, cognitive impairment (CI) and dementia experienced by this population, and culminates in the high rates of early death seen in PEH.^7 8^ Despite a clear public health need for further research, PEH are often not recruited to clinical research, limiting the extent to which the factors contributing to their increased morbidity and mortality can be investigated and addressed.^9 10^

Experiencing homelessness is highly stigmatising, with negative effects on PEH’s mental and physical health.^11^ PEH experience high rates of physical and psychological trauma, which is often recurrent, and often predates their first experience of homelessness.^12^,^15^ This experience of trauma is associated with high rates of complex post-traumatic stress disorder in this group.^16^ The impression that PEH demonstrate complex and difficult behaviours, coupled with their inherent housing instability, leads to a perception that individuals experiencing homelessness are difficult to ‘reach and retain’ in clinical research studies.^9^ Moreover, PEH have high rates of lower literacy levels due to early withdrawal from education, expulsion and suspension, in addition to high rates of underlying intellectual disability.^17^,^21^ Failure to accommodate study participants with lower literacy levels can result in unnecessary exclusion of PEH from participating in research.^9 10^

Recent attempts to improve the recruitment and engagement of PEH in clinical research include the publication of strategies to assist in the engagement of PEH in interventional research studies.^9^ Frameworks to assist researchers in the integration of trauma-informed practices into research design have been developed to improve the inclusion of ‘disadvantaged’ populations in health and social care research.^22^ Additionally, the role of the research nurse in supporting PEH as participants in research has been described.^23^ Moreover, the success of some studies has demonstrated that longitudinal follow-up in a population of PEH can also be feasible.^24^

Homelessness and international migration is increasing worldwide: it is, therefore, imperative that high-quality research is performed to improve health outcomes among PEH.^25 26^ Here, we challenge the perception that recruitment of PEH into clinical research is significantly more challenging than the general population and provide practical strategies based on multistudy experience for enhancing engagement of PEH in public health research. It is the experience of the Inclusion Health Research Group (IHRG) that PEH can be engaged and retained in clinical research studies with a small number of practical supports.^9^ This research augments the existing literature on strategies that have been developed to improve recruitment and retention of PEH into clinical research, by describing the engagement of PEH in observational clinical research by a multidisciplinary specialist clinical research group.

## Methods

Data were collected from researchers and people with lived experience on their experience in the design and delivery of three observational clinical studies recruiting PEH as participants in Dublin, Ireland, between 2021 and 2025. These studies comprise:

(1) Assessing the Effects of Socio-economic status and psychological Stress on Vaccine-induced immunity to SARS-CoV-2 (AESS-Vax); (2) Premature Ageing in long-Term Homeless adults (PATH); (iii) Cognitive Impairment in People experiencing long-term Homelessness, Evidence and Relationships (CIPHER) over 4 years. In total, these studies recruited 178 participants who had experience of homelessness. Participants were recruited from primary care centres providing care to PEH (AESS-Vax) or hostels for PEH (PATH and CIPHER). All three studies involved a blood sample at recruitment, completion of a questionnaire on demographics and health, and completion of several scales (online supplemental document 1, tables 1–3). Participants in the three studies are described in table 1.

**Table 1:** A summary of the study completion and demographic characteristics of the participants recruited into the AESS-Vax, PATH and CIPHER research studies

Characteristic	AESS-Vax, PEH* (n=30)	AESS-Vax, housed (n=118)	PATH (n=63)	CIPHER (n=85)
Study recruitment dates	January 2021 to April 2022	January 2021 to April 2022	January 2022 to August 2023	March 2024 to May 2025
Study completion rate, n (%)	25 (83.3)	118 (100)	59 (93.7)	80 (94.1)
Age, mean (SD)	47.2 (13.28)	40.4 (12.02)	49.8 (10.32)	43.2 (14.00)
Sex, n=male (%)	14 (56.0)	43 (36.4)	45 (71.4)	53 (62.4)
Duration of homelessness, mean, (SD)	Not recorded	N/A	17.5 (12.83)	8.3 (9.10)
Maximum educational attainment, n (%)	Third level: 1 (4.0)	Third level: 81 (68.6)	NFQ level 7: 2 (3.2)	Third level: 10 (11.8)
Current non-prescribed drug use, n (%)	4 (16.0)	3 (2.5)	16 (25.4)	38 (44.7)
Current alcohol use (units per week), mean (SD)	14.4 (13.79)	6.36 (6.67)	52.6 (90.79)	28.8 (50.2)
Smoking pack-year history, mean (SD)	12.8 (10.32)	8.9 (14.19)	33.9 (30.67)	23.2 (32.7)

* Summary statistics for the AESS-Vax PEH cohort are based on participants who completed all study questionnaires and provided at least one blood sample collected (n=25). Scheduling and participant attendance at subsequent planned timepoints were both significantly impacted by ongoing and varying pandemic-related lockdown regulations, and as such granular detail has not been provided.

AESS-Vax, Assessing the Effects of Socio-economic status and psychological Stress on Vaccine-induced immunity to SARS-CoV-2; CIPHER, Cognitive Impairment in People Experiencing long-term Homelessness: Evidence and Relationships; NFQ, National Framework of Qualifications; PATH, Premature Ageing in long-Term Homeless adults; PEH, people experiencing homelessness.

Recruitment to the AESS-Vax study commenced in early 2021, with recruitment closing in April 2022. Study participants fitted into one of three groups, (1) PEH and social exclusion (recruited through the SafetyNet-HSE Inclusion Health Hub, Summerhill, Dublin), (2) People with third level education or higher, not in homelessness and in full time employment (termed ‘high SES’ for the purposes of the study), or (3) People with second level education or less and not experiencing homelessness (termed ‘low SES’). All participants received the BNT162b2 vaccine against SARS-CoV-2. In AESS-Vax, study participants contributed at minimum: a blood sample at recruitment, an initial demographic and health questionnaire and a psychosocial stress and trauma profiling exercise (Perceived Stress Scale,^27^ Holmes-Rahe Life Event Stress Inventory,^28^ Childhood Trauma Questionnaire,^29^ MacArthur’s Ladder).^30^ Participants were invited for follow-up blood draw and data collection at a variety of postvaccination timepoints (4–8 weeks, 12–16 weeks, 22–26 weeks, 48–54 weeks as well as 4–8 weeks and 12–16 weeks post third ‘booster’ dose). Initial recruitment of participants experiencing homelessness was completed in a vaccine clinic at the SafetyNet Inclusion Health Hub, with follow-up blood draws in the St James’ Hospital-Wellcome Trust Clinical Research Facility.

The PATH study commenced in 2022, with recruitment of participants residing in hostels for adults in long-term homelessness in Dublin City between early 2022 and mid-2023. Participants in homelessness completed health and demographic questionnaires with the help of trained researchers. Blood samples and brief health assessments were completed with support from a clinician or research nurse at a single timepoint. Housed controls for this study were drawn from an established biobank within the IHRG/Social Immunology Lab in Trinity College Dublin. The cohort experiencing homelessness recruited here differed from the AESS-Vax cohort experiencing homelessness as it only includes individuals in extreme long-term homelessness, defined here as having experienced 5 or more years in homelessness over their life-course.

Finally, the CIPHER study involved recruitment of adults experiencing long-term homelessness from hostels and day centres serving adults in long-term homelessness in the community from March 2024 to May 2025. In the CIPHER study, long-term homelessness was defined as ≥6 months of homelessness, consistent with the definition used by the Irish Government’s Department of Housing.^31^ Recruitment was followed by the completion of a 2–3-hour cognitive assessment, blood sampling, blood-pressure measurement and grip strength assessment. Participants were invited to attend an additional assessment comprising MR brain imaging and repeat blood sampling. All CIPHER study elements, barring MR imaging, were completed in the community, if that was the participant’s preference. When participants were unable to complete their first assessment in a single sitting, they were invited to complete this at a time and date of their choosing within the following 3 months.

### Public and patient involvement

AESS-Vax and PATH engaged people with lived experience of homelessness during the design of the studies through scheduled meetings with Homeless Health Peer Advocates, during which study aims and objectives, recruitment strategies and assessment tools were discussed. Feedback was incorporated into study design, recruitment and consent procedures and assessments. In CIPHER, a study-specific public and patient involvement (PPI) group of five people with lived experience of homelessness was formed, and a programme of regular, subsidised meetings was devised. These meetings commenced early in the initial study design phase, and PPI contributors provided expertise formed through their lived experience on the study design and recruitment methodology, the location of assessments and the measures employed. Additionally, one of the coauthors of this study with lived experience was instrumental in the design and reporting of this multistudy experience synthesis. Finally, we will disseminate findings of this evidence synthesis via collaborating gatekeeper organisations, comprising service providers for PEH and the SafetyNet Inclusion Health Hub, including accessible summaries for staff and service users.

### Experience synthesis

Members of the IHRG responsible for participant recruitment and engagement in these studies (the study research nurse, principal investigators and PhD researchers), in addition to an expert with lived experience of homelessness, met to evaluate the lessons learnt and experience gained during the recruitment and engagement of PEH in the above-described observational research studies. This process was informed and supported by a review of the literature, which involved exploratory searches of major scholarly databases including PubMed and Google Scholar, and review of key articles identified through reference lists and author expertise. The review was not intended to be exhaustive or systematic, but to inform study design and interpretation. Themes were developed using an iterative team-based process during structured team meetings. Team members who were directly involved in recruitment and participant engagement across the three studies reflected on recruitment/retention barriers and enablers, and these reflections were discussed iteratively with the inclusion of an expert-by-experience. Candidate themes were thereby generated, refined and consolidated across discussions, with agreement reached by consensus. This paper, cowritten with an expert-by-experience, summarises themes extracted from the collaborative discussion and is designed to support future research involving PEH.

## Results

Demographic characteristics and study completion rates of the PEH recruited to date in the three studies are summarised in table 1, with housed participants in AESS-Vax included for comparison. All studies recruited predominantly male participants with an average age in their mid-40s, which is broadly representative of the population experiencing homelessness in Ireland.^32^ Current non-prescribed drug use was reported in 16%–46% of PEH across the three studies, with high levels of alcohol intake per week (14.4 to 52.6 units) and smoking pack-years (12.8 to 33.9) reported.

Following a structured evaluation and discussion, eight overarching themes were identified and summarised as follows:

### The involvement of people with lived experience of homelessness in study design from its inception is paramount

Involving people with lived experience of homelessness from early in the study design phase is integral to the success of research aiming to recruit PEH. Engaged PPI requires active consideration of the power imbalance between the researchers, PPI representatives and study participants. Engagement of people with lived experience ensures that their priorities are considered prior to and during study design. It also ensures that recruitment strategies, the consent process and study assessments are acceptable to the target participants and thereby assists in the maximisation of recruitment. Moreover, PPI contributor review of recruitment and assessment materials ensures that challenges related to literacy, numeracy and reading comprehension that may exist in the target population are considered and addressed.

### Recruitment location should be flexible and recognise the needs and experiences of the participant over the convenience of the study team

Offering to recruit and assess study participants either in the community in their accommodation or day service, or at the time at which they are receiving scheduled medical care significantly decreases the logistical burden placed on the potential study participant by their involvement in the study. The onus is thereby placed on the research team to travel to the participant, removing financial (eg, bus fare to travel to the research site) and cognitive (eg, the researcher must plan the route to the participant, not vice-versa) barriers to participation. Working in collaboration with service providers may support recruitment and assessment of participants in their desired location by allowing the research team to access service 1:1/key-working rooms or private offices. Where a suitable private space is unavailable in the community, providing transport free-of-charge for the participant to travel to a more suitable assessment location should be provided.

Furthermore, offering to perform recruitment and assessment of the participant in the community, where the participant may be more comfortable, rather than in a hospital or university space, serves to reduce the power imbalance between the participant and researcher, improving the tolerability of study participation. Many PEH have had negative experiences when accessing healthcare^33^ and in education^34^ and as such performing study visits in hospital or university spaces may serve as a deterrent to participation in this group.

### Recruitment times should be flexible to suit the priorities and schedule of the study participant

Flexible recruitment and assessment times are important when attempting to maximise recruitment and engagement of PEH in observational research. Scheduling study visits at a time and date suggested by the participant, considering their priorities and schedules, rather than that which best suits the research team has been found to be beneficial. This also aligns with the principles of trauma-informed care by redistributing choice and control.^35^ Providing day-before and/or day-of message reminders to participants and/or their support workers about their appointment, along with penalty-free opportunities to reschedule, is also supportive. Utilising encrypted web-based platforms such as ‘WhatsApp’ to communicate with the participant via message/telephone rather than traditional text message may be useful to keep in contact with participants, as these platforms can be accessed when the participant is connected to Wi-Fi, which is often available even in the absence of phone credit. Giving participants the opportunity to pause their assessments for refreshments or cigarettes, with flexible opportunities to recommence, improves the tolerability of study visits.

Providing direct contact details for the study team, for example, a study-specific mobile phone number and email, rather than scheduling appointments via an intermediary demonstrates respect for the participant and facilitates easy rescheduling of appointments by the participant, or by their representative or keyworker, when necessary. Where assessments in a hospital or research setting are required, providing direct contact details, subsidising and arranging travel, and allowing for rescheduling as needed, maximises participation rates.

### Building trust and setting expectations is necessary to recruit study participants

Building trust between PEH and the research team through consistent follow-through and demonstration of reliability by the study team is important when maximising recruitment and engagement. Trust is built by the research team attending scheduled visits with participants reliably at the agreed time, date and location; giving participants ways to contact the research team easily and consistently as needed; providing promised follow-up on the schedules promised; and through open disclosure when this is not possible. This demonstrates clearly to the study participant that their time and input are valued by the research team.

A robust consent process is necessary not only to ensure that informed consent is obtained prior to participation but also to set participant expectations about what is involved in the study process from their first contact with the research team. In addition to codesign of study research materials with a PPI group with lived experience, and ensuring that these are written to a 10– to 11-year-old reading level to improve inclusivity, providing participants with the option to have the participant information leaflet and consent form read aloud to them is helpful to ensure that they have received appropriate information about the study. Employing a teach-back method is useful to ensure that participants have an understanding of the study and allows them to demonstrate that they have the capacity to retain, weigh and communicate their decision to take part in the research in a sufficiently informed manner.^36^

### Collaboration with gatekeeper organisations and peer advocates supports recruitment and ongoing study engagement

Collaboration with gatekeeper organisations, for example, service providers for PEH, support workers and peer advocates, supports recruitment, particularly at the beginning of the process when participants are identified and first approached. Working with individuals and organisations who have established trust with potential participants helps to build trust with the research team. Holding informal information sessions with the support of the gatekeeper organisations allows both staff involved in recruitment and potential participants to become familiar with the research team in a relaxed, low-pressure environment. These sessions also provide an opportunity to explore what participation in the research involves, while helping to ensure that individuals feel under no obligation to take part if they do not wish to.

Additionally, gatekeepers or peer supporters can be a valuable conduit for communication between participants and the research team, conveying information in an accessible and effective manner, supporting continued engagement by ensuring that participants are prepared to take part in assessments when required. Specifically, this can include providing assessment reminders and facilitating communication between the participant and the research team when the participant is unable to do so, for example, due to a lack of phone credit or access. Peer advocates with direct experience of homelessness are particularly helpful in bridging power imbalances between researchers and PEH and developing trust between the participant and the research team.

### Non-financial incentivisation is beneficial to recruitment

Incentivisation of participants to engage in research reflects that the participant’s time is valuable, but the ethics of providing financial incentives to participation for PEH are complex. It is the policy of our local research ethics committee that no study participant (experiencing homelessness or housed) receives financial incentivisation to participate in research, although compensation for expenses (eg, train tickets) is permissible. However, we have had considerable success in incentivisation of participation through the integration of health-screening elements (eg, blood-borne virus screening), which may otherwise be challenging for the participant to access due to financial constraints. Health screening paired with timely, free clinical follow-up was successfully integrated into the study protocols and well received by participants, ensuring that involvement was not only valuable to the research but also directly beneficial to their health.

### Appropriate selection of assessment tools and the provision of aids

Where available, use of assessment tools designed for and/or validated in populations with experience of homelessness is helpful. Moreover, the provision of aids to assist with participants’ completion of tools, where appropriate, can augment response rates. For example, offering participants access to surveys via links or on tablets to facilitate independent completion by the participant can improve response rates when asking sensitive questions (eg, those contained within the Childhood Trauma Questionnaire).^29^ This can be further improved by providing participants with the option to listen back to questions posed, responses on the tablet, and their answers to support those with lower levels of literacy. Engagement with PPI representatives is critical to identify possibly inappropriate assessment tools, for example, the PPI representatives in CIPHER reported that the National Adult Reading Test was unlikely to be acceptable to participants due to its resemblance to school assessments and, as a result, it was not included in the study protocol.

Participants actively using non-prescribed drugs and alcohol can be recruited and retained in observational studies with appropriate flexibility and support.

As demonstrated in table 1, participants actively using non-prescribed drugs and alcohol were successfully recruited to all three observational studies (CIPHER, PATH and AESS-Vax). Exclusion of participants actively using non-prescribed drugs or alcohol would have resulted in a non-representative study sample and limited applicability of findings. However, it was also important to ensure that intoxication with alcohol or drugs did not affect the consent procedure or study assessments. Given the stigmatisation and potential criminalisation of alcohol and drug use, it was important to specifically consider how this would be assessed to ensure no inadvertent harm was caused to participants. Open, trauma-informed, non-judgmental questions were used to determine participants’ most recent non-prescribed drug or alcohol use. For example, in CIPHER, participants were asked during screening about alcohol and drug use in the preceding 24 hours. The rationale for requiring participants to be non-intoxicated to enable recruitment and participation was clearly explained to participants at this point, as the primary aim of the study involved cognitive assessment, which would be substantially impacted if participants were affected by recent drug or alcohol intake. In the case of a participant reporting recent drug or alcohol use, multiple options to reschedule their review were offered to maximise the participant’s opportunity to engage when sober. Additionally, participants reporting use of alcohol in the preceding 24 hours were asked to undergo breathalysation to check that their breath alcohol levels were under the driving limit (22 μg/100 mL) to ensure that their neuropsychological and cognitive testing results were not unduly affected by high blood alcohol levels. This was acceptable to participants, and all participants complied with this procedure when requested. These strategies ensured that potential participants in active addiction had their opportunity to engage in the research process maximised. However, it is acknowledged that this strategy may result in exclusion of participants with substance use disorder for whom it is necessary to use substances on a regular basis to prevent withdrawal symptoms.

## Discussion

This study reports strategies, aligned with the principles of trauma-informed care that a multidisciplinary group have found to maximise recruitment and retention of PEH in clinical research studies. A perception that PEH are difficult to ‘reach and retain’ often leads to their exclusion from recruitment into clinical research.^9 10^ Here, we show excellent study completion rates, from 83.3% to 94.1%, with experience performing clinical research with PEH that can be applied in future research by other groups to support their inclusion.

Extensive evidence exists on strategies to improve recruitment of housed participants into clinical research. These include open instead of blinded trial designs, the inclusion of opt-out procedures in the study design (instead of taking an opt-in approach), the provision of financial incentives to study participation and the use of telephone reminders to participants in advance of appointments.^37 38^ There is significantly less evidence available when it comes to PEH, and the direct translation of approaches established to be effective in a housed cohort may not be appropriate. The inclusion of opt-out procedures in study design, as opposed to opt-in, may be considered to be less ethically acceptable when applied to a study population with lower health and scientific literacy.^39^ The provision of financial incentives to participation may be similarly ethically ambiguous when applied to a population of PEH, with increased financial vulnerabilities relative to the general population. There is, therefore, a need for bespoke strategies to recruit and engage PEH in clinical, translational and basic health research.

A previous methodology discussion paper described results from a review of the literature for strategies to improve recruitment of ‘harder-to-reach’ populations (among whom the authors included PEH) to qualitative research.^40^ Although this study did not solely focus on PEH, similar themes were identified to those reported here, including the importance of trust, engagement of key stakeholders, recruitment through trusted facilitators and in safe locations.^40^ Although our study was carried out in a single city, the overlap between our findings and those of this international review adds some weight to the generalisability of our findings. Additionally, our study adds to the findings reported previously, providing recommendations specific to PEH, including an emphasis on the importance of PPI, and relevant PPI strategies. Our study also describes strategies to improve recruitment and retention of PEH to observational clinical research, including studies with longitudinal follow-up and involving biological sampling that were not explored by these authors.

Strategies to improve recruitment and retention of PEH with mental illness to interventional research have also previously been published.^41^ The At Home/Chez Soi study was a randomised controlled trial exploring the effects of housing interventions for PEH in Canada. Recruited participants were randomised to receive a Housing First intervention or standard care and were thereafter assessed at four time-points over the following 24 months.^41^ Participants were of a similar age and sex profile to those which we have experience recruiting, and similar recruitment and retention rates were identified following enrolment of PEH to this study, with 79.6% of participants completing four follow-up visits within a 24-month period following recruitment.^41^ Again, some similar themes were identified that supported recruitment of PEH to their study to those which we have described, including the importance of trust, support from gatekeeper organisations and the importance of using tailored study documentation, designed with the input of people with lived experience. The authors additionally describe the use of cash incentives to be ‘probably the most effective tool’ for ensuring that participants remained engaged in the research.^41^ Our study provides novel data supporting the use of non-financial incentivisation to support continued engagement of PEH in clinical research.

The importance of meaningful PPI in research involving PEH has previously been described in improving research quality and relevance, and in helping to address the power imbalance between researcher and participant.^42^ The importance of PPI in participant recruitment and engagement in clinical research in housed populations has been established,^43^ and the experience of the IHRG suggests that it is similarly beneficial when recruiting and engaging PEH.

Ensuring flexibility of study visit location and time in order to prioritise the needs of the study participant over the research team demonstrates the respect and appreciation that the research team has for the participant’s time and input. While not only applicable to PEH, the IHRG believes that this is especially important in this group as they likely have had negative interactions in healthcare settings in the past, which need to be overcome to facilitate their recruitment and engagement in ongoing clinical research.^44^ Recruitment of participants in the community, or at the time at which they are receiving scheduled medical care, reduced barriers to their participation. Cost of transportation to and from the study visit is thereby covered by the study team, reducing participants’ financial barriers to participation. Additionally, there is evidence that PEH experience CI, and in particular executive dysfunction at a higher rate than the general public/housed population.^45^ Recruitment and assessment of participants in the community reduces the impact of potential executive difficulties on their ability to engage with research.^46^

The need to build trust between PEH and the research team is crucial: while this is also important in a housed study population, it is even more essential when working with PEH to overcome previous negative experiences and stigma.^44^ Trust has been additionally leveraged to increase recruitment in research involving PEH through the use of snowball sampling.^47^ Engagement of gatekeeper organisations in the study process is also helpful to build trust between the study team and potential participants. Participants may feel more comfortable liaising with the study team via their support worker, supporting their autonomy when engaging with the research process: participants may feel more able to honestly liaise with the study team via their support worker rather than directly. Recognition of this is crucial to ensuring that participants feel able to engage with freely with the research process, and without undue influence.

In housed populations, financial incentivisation for participation in research is often judged to be ethical, although this is not a consensus judgement.^38^ For PEH, a population that can be considered to be more financially vulnerable than the housed population typically recruited in research, the relative inducement of a financial incentive is significantly higher, and as such could be considered to be unduly influencing a participant’s decision to take part in research. This approach is not universally accepted, and financial incentives have been used in previous observational research in populations experiencing homelessness (in which the risks of participating in the research were judged to be low).^48^ In the experience of the IHRG, offering health screening alongside assertive-engagement outpatient follow-up as a non-financial incentive to participation has been successful in engaging PEH in research studies, especially where this screening would be challenging for the participant to otherwise access, in addition to being ethically favourable relative to a financial incentive.

### Limitations

Limitations of this work are that it summarises experience from a single research group, which has performed research within a single city. The generalisability of the findings may, therefore, be limited, although overlap has been identified with research performed in similar populations in other locations, as previously described. Moreover, although ethics constraints around financial incentives were present in the current research, this may not be applicable in all locations, and the potential additive benefit of financial and non-financial incentivisation for participation has not been explored. Additionally, theme development was via collaborative evaluation rather than a formal qualitative process, which may limit the reproducibility of the study findings. Although themes were agreed by the study authors, formal member checking with study participants was not undertaken. An adapted Delphi process was also not used, which may have been beneficial to reduce the risk of hierarchical influence and to strengthen the reproducibility of the study findings.

## Conclusions

PEH have high rates of morbidity and early mortality yet are often excluded from clinical research. Strategies have been identified that may be beneficial to the recruitment and engagement of PEH in observational research. These include the involvement of robust PPI, flexibility around recruitment location and time, building trust between the research team and participants, collaboration with gatekeeper organisations and the inclusion of non-financial incentives to participation.

## Supplementary material

10.1136/bmjopen-2025-114055 online supplemental file 1

## Data Availability

Data are available upon reasonable request.
