# Supplementary material for: What practical strategies improve recruitment and engagement of people experiencing homelessness in observational clinical research? A multistudy synthesis from Dublin, Ireland
Source: BMJ Open. 2026 Mar 26;16(3):e114055. doi: 10.1136/bmjopen-2025-114055 (PMC13034307; doi:10.1136/bmjopen-2025-114055)
Supplement: online supplemental file 1 [file bmjopen-16-3-s001.docx]

Table 1 A list of psychosocial measures utilised in the AESS-Vax study

| Psychosocial measures used in the AESS-Vax study |
| --- |
| Study-specific questionnaire, capturing demographic and health-related variables. |
| The Perceived Stress Scale |
| The Holmes-Rahe Life Event Stress Inventory |
| The Childhood Trauma Questionnaire |
| McArthur’s Ladder |

Table 2 A list of psychosocial and cognitive measures utilised in the PATH study

| Psychosocial and neurocognitive measures used in the PATH study |
| --- |
| Study-specific questionnaire, capturing demographic and health-related variables. |
| Cantril’s Ladder |
| The Adverse Childhood Experiences Questionnaire |
| A lifetime trauma questionnaire (study-specific) |
| The Clinical Frailty Scale |
| The Barthel Index |
| The Rowland Universal Dementia Assessment Scale |

| Psychosocial and neurocognitive measures used in the CIPHER study |
| --- |
| Study-specific questionnaire, capturing demographic and health-related variables. |
| The Brief Resilience Scale |
| The Ohio State University-Traumatic Brain Injury -Identification Method |
| The Montreal Cognitive Assessment |
| The Rowland Universal Dementia Assessment Scale |
| The Brief Instrumental Functioning Scale |
| The Mental Health Continuum-Short Form |
| The Perceived Stress Scale |
| The Adverse Childhood Experiences Questionnaire |
| The Sydney Language Battery |
| Craft Story-21 Recall |
| The Visual Object Space Perception Battery |
| The Frontal Systems Behaviour Scale |
| The Denis-Kaplan Executive Function System (Verbal Fluency & Colour-Word Interference subtests) |
| The Cambridge Neuropsychological Test Automated Battery (Emotional Bias Task & Spatial Working Memory subtests) |

Table 3 A list of psychosocial and cognitive measures utilised in the CIPHER study.
