# Supplementary material for: What practical strategies improve recruitment and engagement of people experiencing homelessness in observational clinical research? A multistudy synthesis from Dublin, Ireland
Source: BMJ Open. 2026 Mar 26;16(3):e114055. doi: 10.1136/bmjopen-2025-114055 (PMC13034307; doi:10.1136/bmjopen-2025-114055)
Supplement: Supplementary file 2 [file bmjopen-2025-114055.reviewer_comments.pdf]

PEER REVIEW HISTORY

BMJ Open publishes all reviews undertaken for accepted manuscripts. Reviewers are asked to complete a checklist review form and are provided with free text boxes to elaborate on their assessment. These free text comments are reproduced below.

ARTICLE DETAILS

Title (Provisional)

What practical strategies improve recruitment and engagement of people experiencing homelessness in observational clinical research? A multi-study synthesis from Dublin, Ireland

Authors

Richard, Georgia; Reddy, Conor; Lyons, Claire; Argue, Ruth; Bourke, Nollaig M M; Ní Cheallaigh, Clíona

---

VERSION 1 - REVIEW

**Reviewer**	**1**
**Name**	**Buxton, Jane**
**Affiliation**	**The University of British Columbia**
**Date**	**10-Jan-2026**
**COI**	**None**

Interesting paper and topic but needs more clarity re methods

Thank you for the opportunity to review the paper which shares the experience of a research group in 3 studies to provide strategies to improve recruitment of PEH in clinical research. It is an interesting topic and findings are relevant to research with PEH but the method use of how these 8 recommendations were decided upon and validation of this work is unclear and should be further explained and described.

ABSTRACT

Methods

- How were the 3 studies selected? In article it becomes clear these were all undertaken by the same research group and all were in Dublin. This should be made clear in abstract as limits applicability/generalizability of findings and conclusions
- Add dates of studies so can see recency.
- Did authors connect with paper authors for more detail as often much is not included in publications due to word limit?

Results:

States 8 key themes emerged- how were these themes identified – i.e. was this an iterative qualitative process? Were authors of papers engaged?

From list looks like 7! What is missing? Perhaps # 2 is separated out. If numbered would be clearer and less confusing.

1. involvement of people with lived experience in study design;
2. flexible recruitment locations and timings;
3. trust-building through consistent communication;
4. collaboration with gatekeeper organizations and peer advocates;
5. use of non-financial incentives like health screenings;
6. validated assessment tools; and
7. tailored support for participants with

addiction. Main article

METHODS

Here it becomes clear that this study involves one research group in Dublin, Ireland.

Are there any publications from the 3 studies so references and authors can be reviewed? There is a lot of detail about these studies which I found detracted from the study. Simplification of the descriptions and details could be avoided if a protocol or publications could be referenced.

The authors order the studies i) CIPHER; ii) PATH and iii) AESS-Vax but the descriptions are in a different order CIPHER, AESS-Vax then PATH consistency in order would make it flow better

CIPHER, recruitment dates not given. States long-term homelessness but does not define time period. Recruitment from hostels and day centres. Follow-up one or more occasions over max 3 months

PATH, 2022 – mid 2023 hostel residents experiencing long term homelessness (=/>5 years). Please clarify when were participants invited for subsequent follow-up and for what.

AESS-Vax recruitment 2021-April 2022. This study includes 3 subgroups 2 of which do not include PEH so are details regarding those not PEH relevant? Though table 1 showing comparison of those housed completion rate is interesting.

The authors state follow-up at 4-8wks; 12-16 wks; 48-54wks and 4-8wks and 12-16 post 3rd booster. So were there an additional 2 points to receive booster vaccine or were these part of follow-up interactions? What does complete data mean? Please clarify is % completion rate included attendance at all time points i.e. at least 7 occasions? Table suggests 25 attended for at least one blood sample so maybe be different from what reader assumes is study completion.

I suggest Table 1 could include more study details- i.e. dates of enrollment; # of follow-up attended to be considered complete and over what time period from initial enrollment.

The findings seem appropriate and relevant and describe further details but it would help the reader to understand how these categories were determined and named and what/why details

were included in the description. Did they just emerge from the discussion or were they based on literature and evidence? Therefore I suggest expansion of methods.

The authors state “Following a structured evaluation and discussion 8 overarching themes were identified.”

- Was a literature review performed as part of this study? i.e. were the themes based on previous literature
- Was it one person reviewing the study details then bringing it to the group for discussion or was there an iterative process to develop the themes.
- Using an adapted Delphi process can avoid power imbalance between hierarchical and dominant individual pressure especially if including perspectives from PEH and research assistants to reach consensus.
- Did discussion include the named authors or did it also include the research assistants and leads of the 3 studies or perhaps these were the same?
- Was input sought from a number of PEH or just the one co-author?
- Was there any validation or member checking of the themes performed? If so with whom?

PPI is mentioned page 9 of paper line 40 but the term is not previously written in full or defined. It doesn’t even appear 9in the List of Abbreviations on page 17

Page 12 line 12- the authors define Patient Information Leaflet (PIL) but do not use the term again except in their list of definitions

#4 describes need for building trust but in discussion the authors state trust was NOT explored by the HRG

#5 – why is Gatekeeper and Peer Advocates capitalised?

#6 – Congratulations on being able to incentivise participation through non monetary means . I find it worrisome that the studies have been unable to provide honoraria for participants sharing their time and experiences with the researchers. Is this only people who may use substances or for participants of all studies. If so this is very paternalistic and stigmatising and lacks equity.

#8 ‘in addiction’ and ‘addicted to drugs’ may be seen as stigmatizing language. Usually use the term substance use disorder (SUD). However, substances may be used without SUD as a means of survival especially for PEH. Table 1 uses current illicit/non-prescribed drug use.

‘Not under the influence when giving consent’ also seems judgmental, as does ‘need for sobriety’ to be able to participate and only participants ‘engage when sober’. This language seems stigmatizing. It appears potential participants who have a substance use disorder would only be eligible to participate when they had not used for 24 hours and hence were likely in withdrawal. Thus persons experiencing SUD would be excluded.

I found the discussion was more a repeat of results with some references presumably supporting the findings and not a discussion of the study findings and how relates to findings of other previous studies and where similar and how different which would include more of a description of populations involved in other studies to show if generalizable or contrast. Please emphasize what is

new i.e. what this study adds to current knowledge

**Reviewer**	**2**
**Name**	**Mattsson, Elisabet**
**Affiliation**	**Ersta Sköndal Bräcke University College**
**Date**	**20-Jan-2026**
**COI**	**None**

This is a well-written and timely study addressing a highly relevant and often overlooked area i.e., the inclusion of people experiencing homelessness in clinical research. The study provides valuable insights into both structural and practical barriers, as well as meaningful strategies to enhance equity and participation. The paper is clearly organised, and the authors demonstrate strong awareness of the ethical and methodological challenges inherent to this population.

The methods are generally appropriate and sufficiently described to allow replication. The integration of evidence from three observational studies (CIPHER, PATH, and AESS-Vax) is well explained, and the involvement of a multidisciplinary team, including an expert with lived experience, is particularly commendable.

To further strengthen transparency, I would suggest adding a few clarifications:

Please elaborate briefly on how data from the three studies were synthesised (e.g., analytical framework or coding process used).

Clarify inclusion/exclusion criteria and how missing data were handled.

Overall, this is a thoughtful and well-constructed paper that will be of interest to both researchers and practitioners aiming to improve inclusive research design. With minor clarifications, it will make an excellent contribution to the literature on health equity and participation in research.

Recommendation: Minor revision.

VERSION 1 - AUTHOR RESPONSE

Reviewer 1

Thank you for the opportunity to review the paper which shares the experience of a research group in 3 studies to provide strategies to improve recruitment of PEH in clinical research. It is an interesting topic and findings are relevant to research with PEH but the method use of how these 8 recommendations were decided upon and validation of this work is unclear and should be further explained and described.

We thank reviewer one for their time and suggestions and have amended and clarified the manuscript as detailed below.

ABSTRACT

Methods

- How were the 3 studies selected? In article it becomes clear these were all undertaken by the same research group and all were in Dublin. This should be made clear in abstract as limits applicability/generalizability of findings and conclusions.

We have amended the abstract to provide greater clarity about the contextualising studies included in this work. These are three studies conducted by members of the IHRG, from which our experience of recruiting and retaining PEH in observational clinical research is drawn.

- Add dates of studies so can see recency.

Dates have been added to provide additional context.

- Did authors connect with paper authors for more detail as often much is not included in publications due to word limit?

The authors that completed this work are the investigators that carried out the three studies described.

Results:

States 8 key themes emerged- how were these themes identified – i.e. was this an iterative qualitative process? Were authors of papers engaged?

From list looks like 7! What is missing? Perhaps # 2 is separated out. If numbered would be clearer and less confusing.

1. involvement of people with lived experience in study design;
2. flexible recruitment locations and timings;
3. trust-building through consistent communication;
4. collaboration with gatekeeper organizations and peer advocates;
5. use of non-financial incentives like health screenings;
6. validated assessment tools; and
7. tailored support for participants with addiction.

Apologies for this confusion - numbers have been added for clarity to the abstract as suggested. #2 was indeed separated into (2) flexible recruitment locations; and (3) flexible assessment timings. This has been clarified in the text.

Main article

METHODS

Here it becomes clear that this study involves one research group in Dublin, Ireland.

Are there any publications from the 3 studies so references and authors can be reviewed? There is a lot of detail about these studies which I found detracted from the study. Simplification of the descriptions and details could be avoided if a protocol or publications could be referenced.

At the time of submission, full primary publications from the CIPHER, PATH, and AESS-Vax studies are in preparation but have not yet been published. As these manuscripts are currently in preparation, it was not possible to reference peer-reviewed publications at the time of submission.

The authors order the studies i) CIPHER; ii) PATH and iii) AESS-Vax but the descriptions are in a different order CIPHER, AESS-Vax then PATH consistency in order would make it flow better.

This has been amended. The studies are now consistently described in chronological order based on the date of commencement of recruitment (AESS-Vax, PATH, CIPHER).

CIPHER, recruitment dates not given. States long-term homelessness but does not define time period. Recruitment from hostels and day centres. Follow-up one or more occasions over max 3 months.

This has been clarified in the text. Participants were recruited from March 2024 to May 2025. Long-term homelessness in CIPHER was defined as ≥6 months of homelessness, consistent with the definition used by the Irish Government. Participants were assessed on a single occasion in the community and thereafter were invited for MRI and repeat blood sampling. When participants were unable to complete their first assessment in a single sitting, they were invited to complete this at a time and date of their choosing within the following three months.

PATH, 2022 – mid 2023 hostel residents experiencing long term homelessness (=/>5 years). Please clarify when were participants invited for subsequent follow-up and for what.

This has been clarified in the text – PATH participants were assessed at a single timepoint.

AESS-Vax recruitment 2021-April 2022. This study includes 3 subgroups 2 of which do not include PEH so are details regarding those not PEH relevant? Though table 1 showing comparison of those housed completion rate is interesting. The authors state follow-up at 4-8wks; 12-16 wks; 48-54wks and 4-8wks and 12-16 post 3rd booster. So were there an additional 2 points to receive booster vaccine or were these part of follow-up interactions? What does complete data mean? Please clarify is % completion rate included attendance at all time points

i.e. at least 7 occasions? Table suggests 25 attended for at least one blood sample so maybe be different from what reader assumes is study completion.

The % completion rate described for AESS-Vax refers to completion of all study questionnaires and the initial blood sampling. We have not provided data on attendance at follow-up blood sampling as these timepoints were significantly disrupted by ongoing and varying pandemic-related lockdown regulations, and as such further granular detail has not been provided.

I suggest Table 1 could include more study details- i.e. dates of enrollment; # of follow-up attended to be considered complete and over what time period from initial enrollment.

We appreciate this suggestion, and have amended Table 1 to provide additional detail as recommended.

The findings seem appropriate and relevant and describe further details, but it would help the reader to understand how these categories were determined and named and what/why details were included in the description. Did they just emerge from the discussion or were they based on literature and evidence? Therefore I suggest expansion of methods. The authors state "Following a structured evaluation and discussion 8 overarching themes were identified." Was a literature review performed as part of this study? i.e. were the themes based on previous literature. Was it one person reviewing the study details then bringing it to the group for discussion or was there an iterative process to develop the themes. Using an adapted Delphi process can avoid power imbalance between hierarchical and dominant individual pressure especially if including perspectives from PEH and research assistants to reach consensus.

We thank the reviewer for raising this important point regarding transparency in our synthesis methods. Relevant methodological and inclusion-health literature was reviewed to sensitise the team to established approaches and to support interpretation; however, this was undertaken as an integrative/narrative review rather than a formal systematic or scoping review with a pre-registered protocol or standardised search strategy.

Theme development was a team-based process rather than the work of a single individual. Team members who were directly involved in recruitment and participant engagement across the three studies reflected on recruitment/retention barriers and enablers, and these reflections were discussed iteratively in structured team meetings with the inclusion of an expert-by-experience. Candidate themes were generated and refined across discussions, with agreement reached by consensus.

We acknowledge the reviewer's suggestion that an adapted Delphi process could reduce the risk of hierarchical influence and further strengthen reproducibility. This was not undertaken in the current work, and we acknowledge this is a limitation.

Did discussion include the named authors or did it also include the research assistants and leads of the 3 studies or perhaps these were the same?

The named authors were the only researchers involved in the process of participant recruitment and assessment in the described studies. No additional research assistants were involved.

Was input sought from a number of PEH or just the one co-author?

Input from a single co-author with lived experience was obtained for the purpose of this publication, however a public and patient involvement group (PPI group) with lived experience of homelessness provided expert input on the design of the contextualising studies described. This has now been detailed more thoroughly in the study design.

Was there any validation or member checking of the themes performed? If so with whom?

Formal member checking with study participants was not undertaken, representing a limitation of this study. However, validation of themes was supported through iterative discussion among the research team and consensus meetings to refine and agree upon the final themes.

PPI is mentioned page 9 of paper line 40 but the term is not previously written in full or defined. It doesn't even appear 9in the List of Abbreviations on page 17

Apologies - this has been amended, and the list of abbreviations updated.

Page 12 line 12- the authors define Patient Information Leaflet (PIL) but do not use the term again except in their list of definitions

We agree and have amended same.

#4 describes need for building trust but in discussion the authors state trust was NOT explored by the HRG

Thank you for this opportunity to provide clarification - this was not clearly communicated by the study authors. The intended meaning of this sentence was that trust has been leveraged in other research to improve study recruitment through snowball sampling, but snowball sampling has not been explored by the IHRG, nor was it utilised in the described studies.

#5 – why is Gatekeeper and Peer Advocates capitalised?

Thank you for drawing our attention to this – it has been amended.

#6 – Congratulations on being able to incentivise participation through non monetary means . I find it worrisome that the studies have been unable to provide honoraria for participants sharing their time and experiences with the researchers. Is this only people who may use substances or for participants of all studies. If so this is very paternalistic and stigmatising and lacks equity.

It is the policy of our local research ethics committee that no study participant (experiencing homelessness or otherwise) receives financial compensation for study participation, although expenses (such as travel) can be reimbursed. We have clarified this in the text.

#8 ‘in addiction’ and ‘addicted to drugs’ may be seen as stigmatizing language. Usually use the term substance use disorder (SUD). However, substances may be used without SUD as a means of survival especially for PEH. Table 1 uses current illicit/non-prescribed drug use.

‘Not under the influence when giving consent’ also seems judgmental, as does ‘need for sobriety’ to be able to participate and only participants ‘engage when sober’. This language seems stigmatizing. It appears potential participants who have a substance use disorder would only be eligible to participate when they had not used for 24 hours and hence were likely in withdrawal. Thus, persons experiencing SUD would be excluded.

We thank the reviewer for these suggestions and have updated the language used. The rationale for excluding participants that were affected by recent non-prescribed drug or alcohol use in the CIPHER study has been further detailed – the study involved in-depth neuropsychological assessment, the results of which may have been unduly influenced by recent non-prescribed drug or alcohol intake.

I found the discussion was more a repeat of results with some references presumably supporting the findings and not a discussion of the study findings and how relates to findings of other previous studies and where similar and how different which would include more of a description of populations involved in other studies to show if generalizable or contrast. Please emphasize what is new i.e. what this study adds to current knowledge.

Thank you for this suggestion – we have situated this study more clearly in the previously published literature and have aimed to emphasise what is novel in our findings more clearly.

Reviewer 2

This is a well-written and timely study addressing a highly relevant and often overlooked area i.e., the inclusion of people experiencing homelessness in clinical research. The study provides valuable insights into both structural and practical barriers, as well as meaningful strategies to enhance equity and participation. The paper is clearly organised, and the authors demonstrate strong awareness of the ethical and methodological challenges inherent to this population.

The methods are generally appropriate and sufficiently described to allow replication. The

integration of evidence from three observational studies (CIPHER, PATH, and AESS-Vax) is well explained, and the involvement of a multidisciplinary team, including an expert with lived experience, is particularly commendable.

We thank reviewer two for their time and suggestions and have amended and clarified the manuscript as detailed below.

To further strengthen transparency, I would suggest adding a few clarifications:

Please elaborate briefly on how data from the three studies were synthesised (e.g., analytical framework or coding process used).

Data from the three studies were synthesised using a team-based, experience-informed approach rather than a formal qualitative coding framework. The synthesis drew on the direct involvement of the authors in recruitment, consent, follow-up, and participant engagement across all three studies.

Brief summaries of each study's design and completion outcomes are provided to contextualise these experiences. Themes were developed through iterative multidisciplinary discussion, including input from an expert-by-experience, with themes refined and agreed by consensus. This approach was chosen to distil practical, operational lessons across studies rather than to generate theory through primary qualitative analysis.

We have clarified this synthesis process in the Methods section.

Clarify inclusion/exclusion criteria and how missing data were handled.

We thank the reviewer for this suggestion. As this manuscript is not intended to be the primary report of findings from the AESS-Vax, PATH, and CIPHER studies, full inclusion and exclusion criteria and detailed missing data procedures are not presented, as these will be reported in detail in the primary publications currently in preparation. However, a brief description of the eligibility criteria for each study has been included. Additionally, given that this paper only includes a brief summary of study completion rates and participant characteristics, no missing data were present for these variables.

Overall, this is a thoughtful and well-constructed paper that will be of interest to both researchers and practitioners aiming to improve inclusive research design. With minor clarifications, it will make an excellent contribution to the literature on health equity and participation in research.

We hope that these revisions address the reviewers' and editors' comments and have strengthened the clarity and transparency of the manuscript.

VERSION 2 - REVIEW

**Reviewer**	**1**
**Name**	**Buxton, Jane**
**Affiliation**	**The University of British Columbia**
**Date**	**25-Feb-2026**
**COI**	

Thank you for the opportunity to review this revised version of the paper. The authors have addressed both the reviewers' comments thoughtfully and thoroughly. The methods are more transparent and reproducible.

Minor typo

Abstract results – Study completion rates were highT

Strengths and limitations
I suggest much of the findings were previously known e.g. At Home Study, so the study supports existing evidence as well as addresses some gaps.

It is a real trength of the initial studies to engage people with lived experience of homelessness in the design of the studies and creating a PPI group. However, only one person with lived experience was involved in this study and the synthesis of lessons learned and experience with recruitment and engagement, which may not give a full perspective as only one person and no input, validation/member checking of PEH with diverse experiences (age, gender, substance use, length of time experiencing homelessness etc)

Table 1
Please include abbreviations as a foot note, so the table is complete on its own including study names, N/A, SD and NFQ.

8. Participants.
I still find this section stigmatising 'requiring participants sober enough to ….' People who are homeless and have a substance use disorder would likely be excluded as may need to use substances to be able to participate as may experiencing withdrawal. Perhaps another limitation?

VERSION 2 - AUTHOR RESPONSE

Thank you for the opportunity to review this revised version of the paper. The authors have addressed both the reviewers' comments thoughtfully and thoroughly. The methods are more transparent and reproducible.

Minor typo

Abstract results – Study completion rates were highT

Apologies – this has been amended.

Strengths and limitations

I suggest much of the findings were previously known e.g. At Home Study, so the study supports existing evidence as well as addresses some gaps.

It is a real strength of the initial studies to engage people with lived experience of homelessness in the design of the studies and creating a PPI group. However, only one person with lived experience was involved in this study and the synthesis of lessons learned and experience with recruitment and engagement, which may not give a full perspective as only one person and no input, validation/member checking of PEH with diverse experiences (age, gender, substance use, length of time experiencing homelessness etc).

Thank you for these comments – I have amended the Strengths and Limitations section accordingly.

Table 1

Please include abbreviations as a foot note, so the table is complete on its own including study names, N/A, SD and NFQ.

This has now been included.

8. Participants.

I still find this section stigmatising 'requiring participants sober enough to ….' People who are homeless and have a substance use disorder would likely be excluded as may need to use substances to be able to participate as may experiencing withdrawal. Perhaps another limitation?

Thank you for your thoughtful suggestions – this is indeed a limitation of our recruitment strategy, and I have now acknowledged this in the text.
